# 

**Published:** 1853-04

**Authors:** 


### CONTENTS.

### JOURNAL

Page.

ARTICLE I.—Saliva. Its Chemical Constitution and Physiological Function. By A. Snowden Piggot, M. D., Professor of Anatomy and Physiology, Washington University, . . . . 337

ARTICLE II.—Remarks on the Mortality of Children as the Result of Various Sources of Irritation from the First Dentition. By J. L. Levison, D. D. S. . . . . . . . . . . 360

ARTICLE III.—Valedictory Address delivered before the Graduating Class of the Baltimore College of Dental Surgery. By Philip H. Austen, A. M., M. D. . . . . . . . . . . 370

ARTICLE IV.—Gold, Gold-beating, Compounds and Alloys. By Professor R. N. Wright, M. D. . . . . . . . . 387

ARTICLE V.—Dental Patents. By A. Hill, D. D. S., Norwalk, Ct. 400

ARTICLE VI.—American Dental Patents. By Geo. W. Kendall, Cincinnati, Ohio, . . . . . . . . . . . 414

ARTICLE VII.—Daily Jottings in the Gold Book of a Dentist. By E. Townsend, D. D. S., M. D. . . . . . . . . 421

ARTICLE VIII.—Treatment of Hemorrhage. By F. L. Crane, D. D. S., Easton, Pa. . . . . . . . . . . . 428

ARTICLE IX.—Inflammation of the Pulps of Ten Teeth. By John Bilisurio, Dentist. Sydney, Australia, . . . . . 430

### SELECTED ARTICLES.

ARTICLE X.—Retro-Pharyngeal Abscess—Its Medical History and Treatment. By Charles M. Allen, M. D., Resident Surgeon of the New York Hospital, . . . . . . . . . 432

ARTICLE XI.—Purple of Cassius, . . . . . . . . 441

ARTICLE XII.—Tic Douloureux, seated in the Mental Branch of the Inferior Dental Nerve; Division of that Branch; Recovery. Under the care of Mr. Fergusson, . . . . . . . . 443

ARTICLE XIII.—The Structure and Development of Bone. By John Tomes, F. R. S., Surgeon Dentist to the Middlesex Hospital, and Campbell De Morgan, Surgeon to the Middlesex Hospital, 447

ARTICLE XIV.—New Styptic, . . . . . . . . . . 454

ARTICLE XV.—On Exostosis and some other Diseases of the Teeth and their consequences, . . . . . . . . . 456

ARTICLE XVI.—Bony Tumor of the Upper Jaw—Amputation, . 457

ARTICLE XVII.—Death from Hemorrhage from Lancing the Gums, 461

ARTICLE XVIII.—Amputation of a Healthy Upper Jaw, as a preliminary step to the Removal of a Fibro-plastic Tumor of the Pharynx, . . . . . . . . . . . . 462

ARTICLE XIX.—Use of Anæsthetic Agents in Ancient China, . 462

ARTICLE XX.—An Account of a Horn, six and three quarter inches in length, developed upon the Head of a Woman. By Austin L. Sands, M. D., of Cold Spring, N. Y. . . . . . 463

ARTICLE XXI.—Cases of Immobility of the Inferior Maxillary Bone, produced by the abuse of Mercury. By H. H. Toland, M. D., of San Francisco, California, . . . . . . . . 465

ARTICLE XXII.—Impending Death by Chloroform; Resuscitation by raising the Epiglottis, and direct Insufflation, . . . . 474
ARTICLE XXIII.—On the Operation for Cleft Palate. By John Gay, Esq., F. R. C. S., Surgeon to the Royal Free Hospital, . 475
ARTICLE XXIV.—On Stomatitis Materna. By William H. Byford, M. D., Professor of Theory and Practice of Medicine in Evansville Medical College, . . . . . . . . . . 479

### QUARTERLY SUMMARY.

Caries of the Teeth, . . . . . . . . . . . 489
Filling Teeth, . . . . . . . . . . . . . 490
Springing of Plates, . . . . . . . . . . . 490
Extraction of Teeth with the Key, . . . . . . . . 491
The Teeth as objects of Study, . . . . . . . . . 492
Filing the Teeth, . . . . . . . . . . . . 495
A "New Form" of Arsenic for Destroying Dental Nerves and Obtunding the Sensibility of Dentine, . . . . . . 496
Hemorrhage from the Gums, . . . . . . . . . 496
Hemorrhage from the Extraction of Teeth, . . . . . . 496
Death from Hemorrhage, consequent upon Lancing the Gums, . 496
Inserting Pivot Teeth, . . . . . . . . . . . 497
Removing Teeth from Plates, . . . . . . . . . 498
Treatment of Erosion of the Enamel, . . . . . . . 498
Poisonous Chloroform, . . . . . . . . . . . 498
Dr. N. C. Perkins on the subject of Fusel Oil, . . . . . 499
Recipe for Diseased Gums, Cleansing the Mouth, &c. . . 500

### ALUMNI OF DENTAL COLLEGES.

Proceedings of a meeting of Associated Alumni of Dental Colleges, 500

### BIBLIOGRAPHICAL.

Principles of Physiology, General and Comparative, . . . 502
Demonstration of Anatomy, . . . . . . . . . 502
A Manual of the Dissection of the Human Body, . . . . 503
Valedictory Address, . . . . . . . . . . . . 503
Introductory Lecture, . . . . . . . . . . . 503
Introductory Address, . . . . . . . . . . . 503
A Manual of Human Anatomy, . . . . . . . . . 504
Southern Journal of the Medical and Physical Sciences, . . 504

### EDITORIAL DEPARTMENT.

Annual Commencement of the Baltimore College of Dental Surgery, 505
Commencement of the Philadelphia College of Dental Surgery, 507
Commencement of the New York College of Dental Surgery, . 507
American Academy of Dental Science, . . . . . . 508
Dental Education, . . . . . . . . . . . . 509
Prize Essay, . . . . . . . . . . . . . 510
Ossification of the Dental Pulp, . . . . . . . . 510
Transposition of Teeth, . . . . . . . . . . . 510
Dr. Hullihen's Method of Mounting Single Teeth, . . . . 510
The Virginia Medical and Surgical Journal, . . . . . 510

